# Undesirable immigrants: hobbyist vivaria as a potential source of alien invertebrate species

**DOI:** 10.7717/peerj.7617

**Published:** 2019-09-17

**Authors:** Radomir Jaskuła, Anna Sulikowska-Drozd, Aleksandra Jabłońska, Krzysztof Banaś, Tomasz Rewicz

**Affiliations:** 1 Department of Invertebrate Zoology and Hydrobiology / Faculty of Biology and Environmental Protection, University of Lodz, Łódź, Poland; 2 Department of Plant Ecology / Faculty of Biology, University of Gdansk, Gdańsk, Poland

**Keywords:** Exotic pets, DNA barcode, Isopoda, COI, Gastropoda, Turbellaria, Annelida, Alien species, Myriapoda

## Abstract

**Background:**

Small size and large diversity of adaptations make invertebrates a group of animals which can be easily transported by different human activities. Many species can travel as “hitchhikers” with plant material (both on plant surfaces and in the soil), including plants used for decoration in vivaria. Vivaria are often tropical in nature environments, with high temperatures and humidity, suitable for invertebrates from tropical regions. Although many of such invertebrates cannot survive in temperate regions where harsh weather conditions are present, it is also known that some can successfully acclimatise. As a result, their negative impact on local flora and fauna cannot be excluded.

**Material and methods:**

Terrestrial invertebrates were collected in several cities of Poland from tropical vivaria where poison dart frogs (Dendrobatidae) and/or orchids (Orchidaceae) were kept by hobbyists. Collecting of the material was preceded by a simple questionnaire placed on the biggest Polish forum devoted to poison dart frogs. Moreover, we contacted some Polish wholesalers offering tropical invertebrates (Isopoda and Collembola), used as the food source for frogs, hoping to receive information about locations where those invertebrates were delivered, over the period of one year. We obtained mtDNA barcodes using the COI marker (cytochrome c oxidase subunit I gene) for seven potential morphospecies.

**Results:**

In total, 12 taxa classified as Turbellaria, Annelida, Gastropoda, Isopoda, Diplopoda, Chilopoda and Collembola were collected and preserved in pure ethanol. We collected material and/or information from 65 locations, including 56 cities to which exotic isopods and springtails were sold by wholesalers over the period of nine months (average number per month = 18 cities). We obtained 18 COI sequences which were assigned to seven BINs and thus confirmed identification of seven species. The results indicate that the number of species transported with exotic plants is not small and can be observed regularly. Species noted as “hitchhikers” on plant structures and/or as inhabitants of soil in plant pots, originally came from South and Central America, Africa, Asia and possibly from North America or Southern Europe. Three taxa were noted for the first time from Poland, including *Rhynchodemus sylvaticus* (Rhynchodemidae), *Trichorhina* sp.1 (Platharthridae), and *Guppya gundlachi* (Euconulidae).

**Discussion:**

The presented study clearly shows that an exotic hobby such as keeping tropical poison dart frogs and/or orchids may promote fast and uncontrolled dispersion of a high number of invertebrates classified in different taxonomical groups. Plant material (green elements of plants and the soil in which they are planted) used in vivaria can be an important source of such animals.

## Introduction

In recent history, the predominant method of alien species dispersion to new regions of the world is connected to human activity (Williamson, 1996; Robinson, 1999; Roques et al., 2009). Many animals, including invertebrates, are regularly transported for long distances, often to different continents, with ballast water or with transportation of exotic fruits (Williamson, 1996; Roques et al., 2009). The majority of such individuals cannot survive under the new and often completely different environmental conditions but some of them can adapt to artificial habitat (e.g.: in greenhouses) where conditions are similar to natural (e.g.: Korkós et al., 2002; Stoev, 2004; Cowie, 2005; Boros, 2011; Kolicka et al., 2015; Kolicka et al., 2016). Moreover some species which are intentionally imported from the country of origin to be kept as exotic pets may play an important role in this process (e.g.: Chucholl, 2015; Jabłońska et al., 2018), as they can be potentially invasive in new areas, or they can be a reason for importing additional material (e.g.: exotic plants used for decoration in “natural habitat-type” terraria or aquaria in which such species are kept). Among such animals there are mainly vertebrates, e.g.: red-eared terrapin (*Trachemys scripta elegans*), originally known from the wetlands of Florida (USA) but now also widely distributed in some regions of Southeast Asia, Southern Europe, South Africa and tropical America (Lever, 2003; Kopecký, Kalous & Patoka, 2013; Pearson, Avery & Spotila, 2015), or mosquito fish (*Gambusia* spp.), originally known from the southern parts of North and Central America and now easy to find in warm waters almost all over the world (Chucholl, 2013; Maceda-Veiga et al., 2013). Many such terrarium/aquarium species can be found in natural sites as a result of intentional introduction by releasing unwanted pets which is often believed to be an ethical solution (Duggan, 2011). In the case of many invertebrates the situation is usually different, as most of them are not intentionally bred in terraria, paludaria and/or aquaria (hereon, termed “vivaria”). Moreover, they are usually accidentally present in such collections, as a result of placing new plants in tanks (Duggan, 2010; Patoka et al., 2015) or because they are used as a source of food for fish, amphibian or reptile pets (e.g.: Cochard, Vilisics & Secher, 2010; McMonigle, 2013). Some of them are known as parasites of reptiles and/or amphibians (e.g.: Kenny et al., 2004; Brianti et al., 2010; Nowak, 2010; Mihalca, 2015) or may be a potential disease vectors (Robinson, 1999). According to Schileyko (2010) and Richling (2017), the diversity of tropical terrestrial snails incidentally introduced to greenhouses in Europe remains yet to be discovered.

The aim of this study is to investigate the significance of private tropical terraria in Poland as a pathway for the introduction of alien invertebrate species and to undertake preliminary estimation of their taxonomic diversity.

## Material and Methods

**Figure 1:**
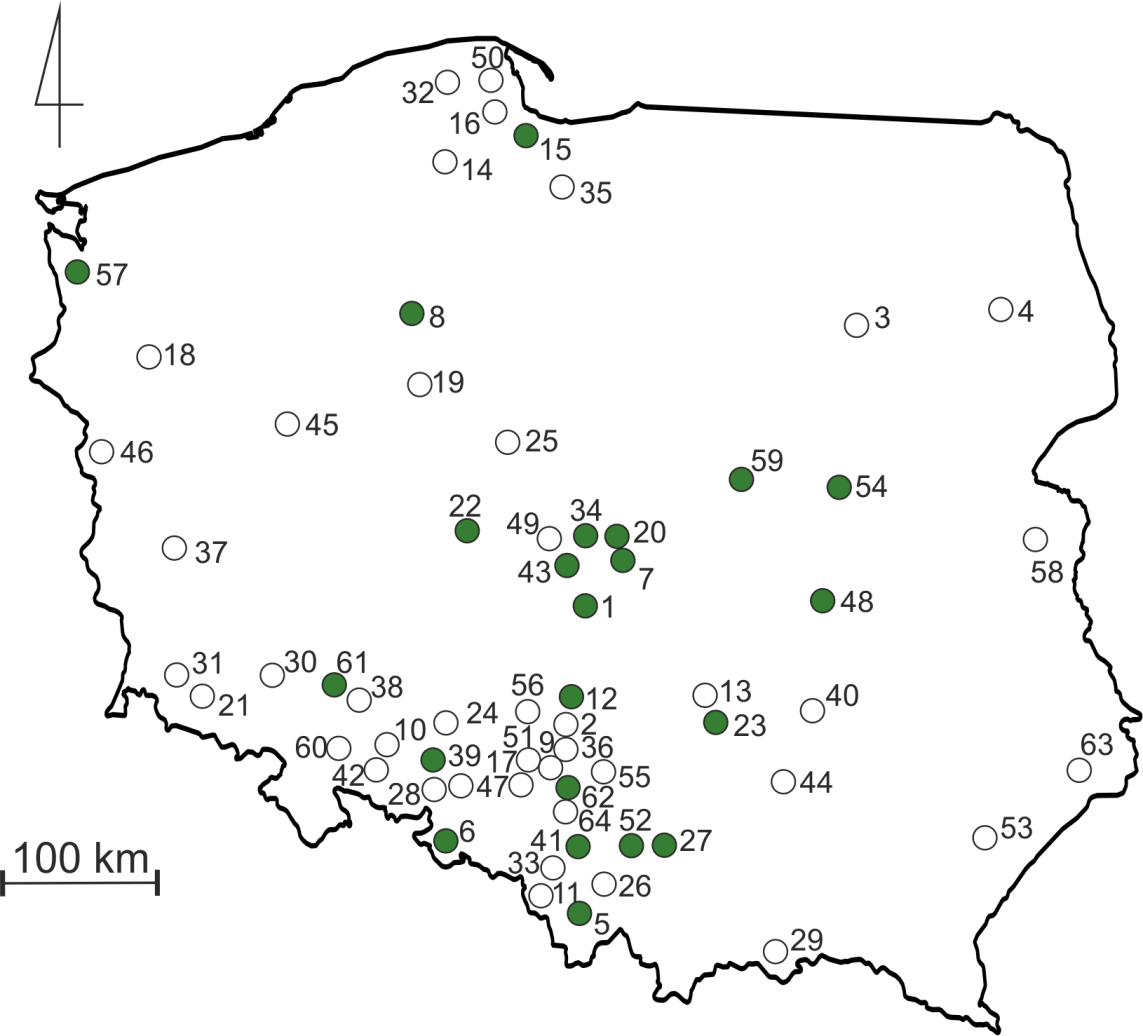
Map of Poland with sampling sites. Green dots indicate terraria locations from where invertebrates were collected. Empty dots indicate locations to which Collembola and Isopoda were sent by suppliers as a food source. Numbers refers to those listed in Table S1.

Terrestrial invertebrates were collected from tropical cultures (vivaria) of poison dart frogs (Dendrobatidae) and/or orchids (Orchidaceae) kept in collections in 22 localities in Poland. All invertebrates were collected by “active searching” (specimens were captured and preserved in pure ethanol for future identification), if possible during few months (“sampling period” was different in different collections). The collecting of material was preceded by a simple questionnaire placed on the biggest Polish forum devoted to poison dart frogs (http://www.drzewolazy.org.pl). Two questions were asked: (1) do you have any invertebrates in your vivarium; and if you do, (2) what was the source of plants used for decoration (if possible, please provide details about the city and/or country of plant origin)? The second question was asked because plants are regularly noted in many European forums focused on tropical amphibians as potential source of undesirable invertebrates. Every person who provided a positive answer for the first question was then asked to collect recorded invertebrates, preserve them in pure ethanol and send for taxonomical analysis at the University of Lodz. To increase the number of collected specimens, we offered tubes and ethanol for preservation of invertebrates and we also offered to pay for sending samples. Moreover, we contacted some of the biggest Polish wholesalers (only two replied: “dendrobates.pl”, “Tropical Factory”) that sell tropical invertebrates (Isopoda and Collembola) as a source of food for poison dart frogs, to receive information about locations where they had sent such animals over the course of the last year (on the basis of received information we noted that both groups regularly are imported from Germany and/or Czech Republic). During the colder winter months, suppliers do not send packages with live invertebrates; therefore, the data received from them refers to the period from March to October. This was used to estimate the number of locations where such alien invertebrates could be sent during one season. As the result it was possible to evaluate the “’human-mediated extent of dispersal”. All the localities of the investigated collections in Poland are presented in Fig. 1 and Table S1.

Investigated specimens (Turbellaria, Annelida, Gastropoda, Isopoda, Diplopoda, Chilopoda, Collembola) were preserved in pure ethanol and later identified (if possible to species level) in the laboratory using traditional keys and, if possible, confirmed by DNA barcodes. All the material used in this study is stored in the permanent collection of the Department of Invertebrate Zoology and Hydrobiology, University of Lodz, Poland.

DNA of Gastropoda was extracted using the Genomic Mini Kit, according to the manufacturer’s protocol. DNA from Turbellaria and Arthropoda was extracted using the standard phenol-chloroform method following Hillis, Moritz & Mable (1996). Air-dried DNA pellets were eluted in 100 µl of TE buffer, pH 8.00, stored at 4 °C until amplification, and subsequently at −20 °C in long-term storage. The cytochrome oxidase subunit I gene (COI; ca. 670 bp long fragment) with LCO1490/HCO2198 primers (Folmer et al., 1994) was amplified. In cases when amplification failed, we used LCO1490 JJ/HCO2198 JJ (Astrin & Stüben, 2008) primers. For both primer pairs, we used PCR reaction conditions following Hou, Fu & Li (2007). PCR products (5 µl) were cleaned up by Exonuclease I (2 U; Thermo Scientific) and alkaline phosphatase FastAP (1 U, Thermo Scientific) treatment, according to the manufacturer’s guidelines, and sequenced directly using the same primers as at the amplification stage. Sequences were obtained with the BigDye sequencing protocol (Applied Biosystems 3,730×l) by Macrogen Inc., South Korea. The obtained sequences were edited, aligned and trimmed with the CLUSTALW algorithm (Chenna et al., 2003) using BIOEDIT©7.2.5.

For verification, BLAST (nBLAST, search set: others, program selection: megablast) (Altschul et al., 1990) and/or BOLD (identification engine; species level barcode records) (Ratnasingham & Hebert, 2007) searches were performed to confirm or compare obtained sequences to those already published.

The sequences already deposited in BOLD Systems database were compared with the newly submitted ones. Afterwards, according to their molecular divergence, the sequences were clustered using algorithms identifying discontinuities between clusters. A unique and specific Barcode Index Number (BIN) was assigned for each cluster database (Ratnasingham & Hebert, 2013). If the submitted sequences did not group together with already known BINs, a new number was created. Each BIN was registered in the BOLD Systems. We analyzed intra- and interspecific distances of the identified species using the provided analytical tools of the BOLD workbench (align sequences: BOLD aligner; ambiguous base/gap handling: pairwise deletion) based on the Kimura 2-parameter model of sequence evolution (K2p; Kimura, 1980). DNA barcodes were compared with other COI sequences of identified species available in BOLD (Table 1) and/or in the GenBank database. Phylogenetic trees (Figs. S1–S7) were constructed in MEGA 6 (Tamura et al., 2013) using the Neighbor-Joining (NJ) method (Saitou & Nei, 1987) based on Kimura 2-parameter model of sequence evolution (Tamura et al., 2013) with a bootstrap test performed on 10,000 replicates (Felsenstein, 1985), for all seven species identified by molecular methods. To generate the trees all sequences belonged to members of each BIN and/or species or genus (case of *Lehmannia valentiana*) as well as sequence of the nearest neighbour were used (Figs. S1–S7).

**Table 1:** BOLD distance analysis of the studied sequences of terrarium invertebrates.

Order	Species	*Gn*	*n*	BIN	nBIN	GenBank accession	MID	DNN	NNS	NJ tree
Tricladida	*Rhynchodemus sylvaticus*	5	4	ADF9762	1	MH295756–MH295759	0.6	17.08	*Platydemus manokwari*	Fig. S1
Isopoda	*Trichorhina tomentosa*	6	4	ADG1585	1	MH295760–MH295763	0.19	19.81	*Gammarus nekkensis*	Fig. S2
Polydesmida	*Oxidus gracilis*	67	2	AAG5070	1	MH295752–MH295753	0.91	13.79	Xystodesmidae	Fig. S3
Collembola	*Coecobrya tenebricosa*	4	2	ACZ2224	1	MH295743–MH295744	0.16	16.01	Entomobryidae	Fig. S4
Stylommatophora	*Zonitoides arboreus*	74	3	AAM0135	6	MH295764–MH295766	2^a^	1.04	*Zonitoides arboreus*	Fig. S5
Stylommatophora	*Guppya gundlachi*	1	1	ADH3853	1	MH295749	–	8.08	*Kaliella scandens*	Fig. S6
Stylommatophora	*Lehmannia valentiana*	17	2	AAJ1995	1	MH295750–MH295751	0.76	8.49	*Lehmannia nyctelia*	Fig. S7

**Notes.**

Gn global number of analyzed specimens n our new data BIN barcode index number nBIN number of BIN in species MID maximum intraspecific pairwise K2P distances DNN minimum interspecific pairwise K2P distances to the nearest neighbor species NNS the nearest neighbor species NJ tree reference to supplementary Neighbor Tree

a Indicate species with higher intra than interspecific K2p distance.

Relevant voucher information is accessible through the public data set “TERIN” (available publicly in BOLD) in the Barcode of Life Data Systems (BOLD; http://v4.boldsystems.org/; Ratnasingham & Hebert, 2007).

## Results

### Diversity of invertebrates in terraria

Data on invertebrates noted from tropical vivaria located in 65 Polish cities were used for study including samples collected from 22 localities by 26 hobbyists who provided positive answers in questionnaire presented on Polish internet forum. The number of places to which Collembola (*Coecobrya tenebricosa*) and Isopoda (*Trichorhina tomentosa*) were sent by wholesalers over the course of nine months was relatively high (in total, 56 Polish cities, with the average number of 18 cities per month). Moreover, one of the wholesalers delivered such invertebrates to additional destinations, in the Czech Republic, Germany and Sweden.

**Figure 2:**
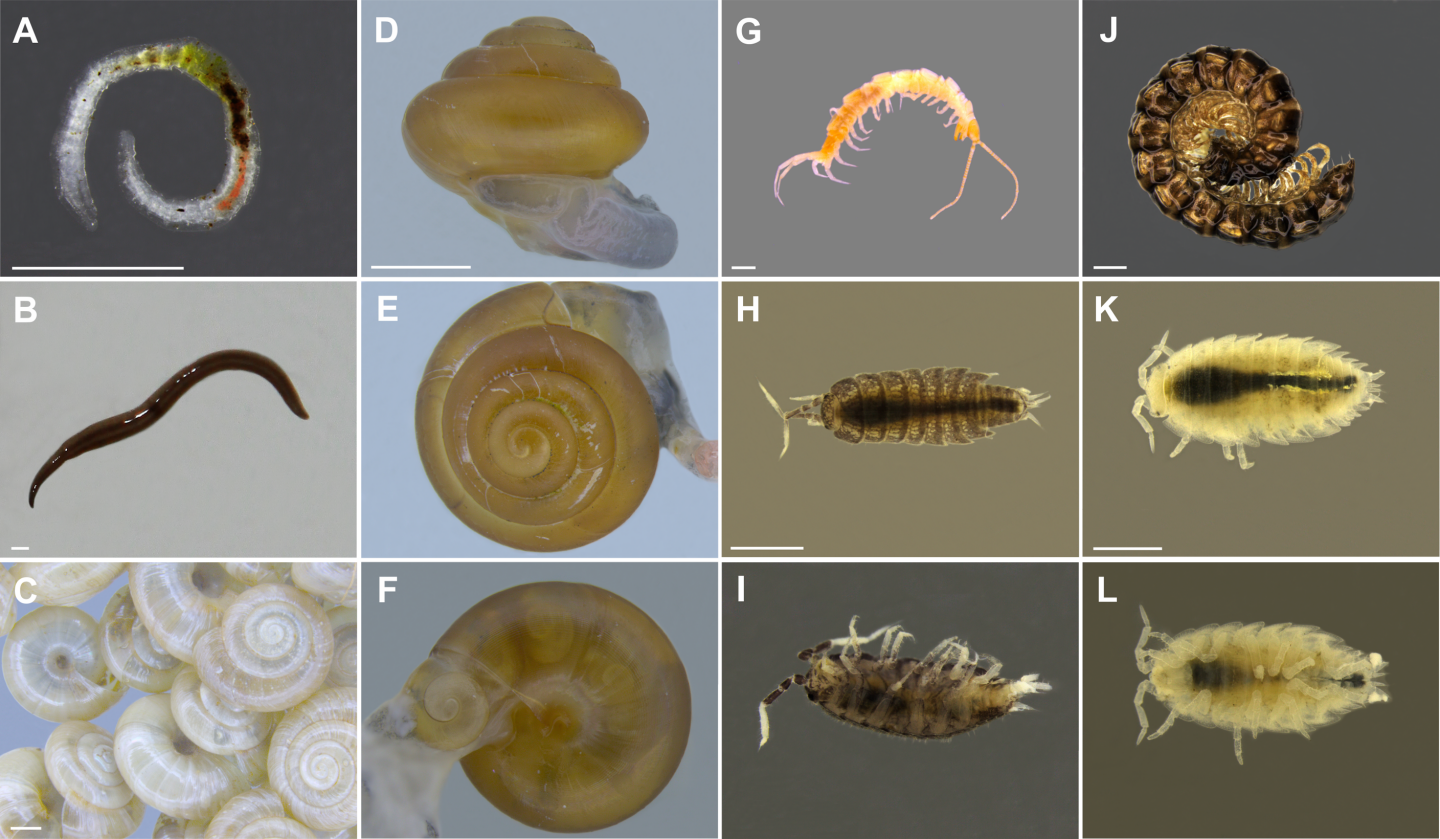
Examples of invertebrates recorded in terraria. (A) Enchytraeidae non. det., (B) *Rhynchodemus sylvaticus*, (C) *Zonitoides arboreus*, (D–F) *Guppya gundlachi*, (G) Lithobiomorpha non. det., (H–I) *Trichorhina* sp.1, (J) *Oxidus gracilis*, (K–L) *Trichorhina tomentosa;* scalebar = 1 mm (phot. A, (G–L) R. Jaskuła, (C–F) A. Drozd).

A total of 12 invertebrate taxa (different taxonomic levels), alien to Polish fauna, and in most cases also to European fauna, were noted. These were classified in four animal phyla including Platyhelminthes (Tricladida), Annelida (Oligochaeta), Mollusca (Gastropoda) and Arthropoda (Collembola, Diplopoda, Chilopoda and Isopoda) (Fig. 2). The list of recorded taxa is placed in the section “Recorded taxa” (numbers indicating localities are referring to the Table S1, Fig. 1).

In case of material collected by hobbyists in their collections, we received information that invertebrates classified as Tricladida, Oligochaeta, Gastropoda, Diplopoda and Chilopoda, started to be seen in vivaria usually soon after new tropical plants were added to the tanks (Bromeliaceae, ferns and/or *Peperomia* spp.); Germany, the Netherlands and Czech Republic were noted as the countries from which such plants were imported. Three taxa, *Rhynchodemus sylvaticus* (Leidy, 1851), *Trichorhina* sp.1, and *Guppya gundlachi* (Pfeiffer, 1840), were reported from Poland for the first time. In two latter cases, the first record was based on the literature data which were compared with morphological characters of our new material. Recorded invertebrates were recognised as originally occurring in South America (two species), Central America (two species), tropical North America (1–2 depending on whether *Rhynchodemus sylvaticus* is accepted as native to this region or not), Africa (one species), Eastern Asia (one species) and Southern Europe (one species). In the case of Lumbricidae, Enchytraeidae, Chilopoda and juveniles of Diplopoda it was not possible to identify species and, as a result, we could not provide details about their original distribution. On the other hand, we cannot exclude that at least in some cases undetermined juveniles of Diplopoda could be classified as *O. gracilis*, as in the same samples adult specimens of that species were present.

Among recorded alien invertebrates, we noted two predators (Lithobiomorpha non. det. and *R. sylvaticus*), three herbivores (*Zonitoides arboreus*, *Guppya gundlachi, Lehmannia valentiana*), and five detritivores (*Coecobrya tenebricosa*, *Oxidus gracilis*, Diplopoda non. det., Lumbricidae non. det., Enchytraeidae non. det.). In the case of *Rhynchodemus sylvaticus*,** we noted predatory behaviour towards fruit flies (*Drosophila melanogaster*) and springtails (on the basis of personal observations). In the case of *Zonitoides arboreus*, we have found that this species regularly feeds on miniature orchids (Orchidaceae), while *Guppya gundlachi* forages on other foliage plants (mainly *Peperomia*).

Overall, 18 mtDNA COI sequences were obtained, which were assigned to 7 BINS in the BOLD Database. Only one of them, assigned to *Guppya gundlachi* (Gastropoda) formed a new unique BIN. The K2p distance to its closest neighbour, *Kaliella scandens* (Cox, 1872), amounted 8.08%. The remaining six species were grouped in earlier established BINs (non-unique BINs) containing from one to 71 sequences already existing in BOLD database (Table 1). Maximum intraspecific pairwise K2p distances within BIN were quite low, ranging from 0.16 to 0.91%. In case of *Zonitoides arboreus* (BIN AAM0135) the intraspecific distance was higher than interspecific, and had a value of 2.0% comparing to 1.04% respectively (Table 1). This gastropod species is assigned to six existing BINs, which may indicate some divergent lineages or even a complex of cryptic species. Summarizing all generated NJ trees and BIN assignments confirmed our species identification (Figs. S1–S7).

### Recorded taxa

Phylum: Platyhelminthes

Order: Tricladida

Family: Geoplanidae

**Table:** 

***Rhynchodemus sylvaticus* (Leidy, 1851)**
**Origin:** Described from the USA, but most probably native to SW Europe (Jones, 1998);
**Actual distribution:** USA (Ogren & Kawakatsu, 1998), France, Spain, Great Britain, Portugal (Álvarez Presas et al., 2014), Bermuda (Jones & Sterrer, 2005), Poland (this paper);
**Localities in Poland:** 1, 27, 34, 39, 62;
**Comments:** In Opole (39) observed as predator on Collembola and wing-less *Drosophila* specimens (J Faron, pers. obs., 2014–2015), confirmed by DNA barcodes (see results of the study). Probably introduced with tropical plants (Bromeliaceae) imported from Germany. **First record from Poland.**

Phylum: Arthropoda

Order: Isopoda

Family: Platharthridae

**Table:** 

***Trichorhina tomentosa* (Budde-Lund, 1893)**
**Origin:** Tropical South America (Araujo & Buckup, 1996; Cochard, Vilisics & Secher, 2010);
**Actual distribution:** Tropical America, North America, Austria, Belgium, Czech Republic, France, Great Britain, Germany, Hungary, Ireland, the Netherlands, Norway, Poland, Switzerland (Cochard, Vilisics & Secher, 2010);
**Localities in Poland:** 1–65;
**Comments:** Used as food for small tropical amphibians and reptiles, and thus regularly kept by hobbyists in high number of individuals. Known as the most widespread alien terrestrial crustacean in Europe (Cochard, Vilisics & Secher, 2010).
***Trichorhina* sp.1**
**Origin:** Tropical America;
**Localities in Poland:** 34, 39, 53, 62–63;
**Comments:** Used as food for small tropical amphibians and reptiles, less popular than previous species, kept by hobbyists in high number of individuals. **Although we were not able to identify the species name, it is evident that this isopod was not recorded from Poland before.**

Class: Diplopoda

Family: Paradoxomatidae

**Table:** 

***Oxidus gracilis* (C. L. Koch, 1847)**
**Origin:** East Asia (Jędryczkowski, 1982);
**Actual distribution:** Cosmopolitan, noted from most continents, in Europe recorded from many countries, mainly from greenhouses (e.g., Hoffman, 1999; Stoev, 2004; Enghoff & Moravvej, 2005; Shelley & Sikes, 2012);
**Localities in Poland:** 5–7, 20, 34, 39, 60;
**Comments:** At least in a few cases came with tropical plants (Bromeliaceae, Orchidaceae) bought from Germany and/or Ecuador (R Jaskuła, K Olszowski, P Tupczański, pers. obs., 2014–2017).
**Diplopodanon. det.** (juveniles)
**Origin:** -
**Actual distribution:** -
**Localities in Poland:** 20, 34, 39;
**Comments:** Came with tropical plants (Bromeliaceae) from Germany (R Jaskuła, pers. obs., 2014–2016).

Class: Chilopoda

**Table:** 

**Lithobiomorpha non. det.**(juveniles)
**Origin:** ?
**Actual distribution:** ?
**Localities in Poland:** 39;
**Comments:** -

Class: Collembola

Family: Entomobryidae

**Table:** 

***Coecobrya tenebricosa* (Folsom, 1902)**
**Origin:** USA
**Actual distribution:** USA, Spain, the Netherlands, Denmark, France (http://www.gbif.org; Zhang et al., 2015), Poland
**Localities in Poland:** 1–65;
**Comments:** Sold as food for tropical frogs, thus probably kept in many other cities of Poland.

Phylum: Mollusca

Class: Gastropoda

Family: Gastrodontidae

**Table:** 

***Zonitoides arboreus* (Say, 1816)**
**Origin:** North and Central America (Evangelista et al., 2013);
**Actual distribution:** North, Central and South America, Asia, Africa, Europe (see: Stworzewicz, 2011a; Evangelista et al., 2013);
**Localities in Poland:** 5, 7, 15, 34, 39, 60, 62;
**Comments:** Came with tropical plants (Orchidaceae) from Germany and/or the Netherlands and/or Ecuador. In Bielsko-Biała (5), Łódź (34), and Warsaw (60) noted as orchid feeder (K Olszowski, R Jaskuła, P Tupczański, pers. obs., 2014–2015).

Family: Euconulidae

**Table:** 

***Guppya gundlachi* (Pfeiffer, 1840)**
**Origin:** Central America (Schileyko, 2002);
**Actual distribution:** North, South and Central America, Caribbean, SE Asia (Thailand) (idtools.org), Poland (this paper);
**Localities in Poland:** 34;
**Comments:** Came with tropical plants (*Peperomia* spp.) (R Jaskuła, pers. obs., 2014–2016). **First record from Poland.**

Family: Limacidae

**Table:** 

***Lehmannia valentiana*******(Férussac, 1822)**
**Origin:** Iberian Peninsula and Northern Africa (Wiktor, 1973; Stworzewicz, 2011b);
**Actual distribution:** Worldwide; in temperate regions only indoors (Stworzewicz, 2011b; Welter-Schultes, 2012);
**Localities in Poland:** 15;
**Comments:** -

Phylum: Annelida

Class: Clitellata

Family: Lumbricidae

**Table:** 

**Lumbricidae non det.**
**Origin:** -
**Actual distribution:** -
**Localities in Poland:** 34;
**Comments:** Came with tropical plants (Bromeliaceae) (R Jaskuła, pers. obs., 2014–2016);

Family: Enchytraeidae

**Table:** 

**Enchytraeidae non det.**
**Origin:** -
**Actual distribution:** -
**Localities in Poland:** 34;
**Comments:** Came with tropical plants (*Peperomia* spp.) (R Jaskuła, pers. obs., 2014–2016).

## Discussion

### Diversity of alien invertebrates in terraria

As a result of the presented study, we have noted 12 invertebrate taxa alien to Polish fauna classified into four different animal phyla. Among species noted by us in this study, three taxa were recorded for the first time from Poland: *Rhynchodemus sylvaticus* (Rhynchodemidae), *Trichorhina* sp.1 (Platharthridae), and *Guppya gundlachi* (Euconulidae). So far *Zonitoides arboreus* (Gastrodontidae) was found only in one greenhouse in Poland, while *Lehmannia valentiana* (Limacidae) in two similar sites (Stworzewicz, 2011a; Stworzewicz, 2011b). Although we have not been able to identify all the material to the species level, we believe that this diversity is high (in terms of origin and feeding strategy), especially that we have noted species deriving from four continents including predators and herbivores. Based on the collected data, we know that quite probably most species/specimens were accidentally imported to hobbyists’ collections with plant material (probably both on plant surfaces and in the substrate in which plants were kept). Although the amount of data from vivarium collections is rather limited, there are other numerous examples of such accidental “hitchhikers” in the case of plants grown in greenhouses (e.g., Korkós et al., 2002; Kaszuba & Stworzewicz, 2008; Boros & Dózsa-Farkas, 2008; Leiss, Reischütz & Reischütz, 2008; Horsák, Juřičkova & Picka, 2013; Evangelista et al., 2013; Zawierucha et al., 2013); or aquaria (e.g.: Duggan, 2010; Patoka et al., 2015). In the case of different taxonomic groups studied before, plants and container shipments were known as the most important source of alien terrestrial invertebrates, including e.g.: mites (Kobelt & Nentwig, 2008), spiders (Niedbała, 2010; Zawierucha et al., 2013), land planarians (Ogren, 1985; Mather & Christensen, 1992; Hogan & Dunne, 1996; Winsor, Johns & Barker, 2004; Justine et al., 2014), snails (Robinson, 1999; Cowie, 2005; Dvořak & Kupka, 2007; Głowaciński et al., 2011; Von Proschwitz et al., 2017), diplopods (Stoev, 2004) or oligochaetes (Boros & Dózsa-Farkas, 2007; Boros, 2011). In addition, only in the case of two (or three) taxa, springtail *Coecobrya tenebricosa* and isopods *Trichorhina tomentosa*, as well as *Trichorhina* sp.1, their presence in terraria is directly connected with the use of those invertebrates as a regular food source for poison dart frogs (Lötters et al., 2007; McMonigle, 2013; Steinmann & Van der Lingen, 2013). Moreover, tropical isopods are also known as important detritivores that keep the collections clean in such husbandries (McMonigle, 2013). As a consequence at least one of these species (*Trichorhina tomentosa*) is widely distributed also out of its native range (Cochard, Vilisics & Secher, 2010).

### Potential “dispersal power” of alien vivarium invertebrates

Most species noted in the presented study have a native range in the tropics (see aforementioned section *Recorded taxa* in Results), where both temperature and humidity are much higher than in temperate regions, including Poland. As a result, we can expect that most of these species can survive only under conditions identical or similar to those observed in their natural habitats, which in Central Europe can be found only in greenhouses, palm houses and collections of tropical frogs or orchids. Thus, their potential dispersal power in Poland and in Central Europe is not high, being strongly limited by climate. As a consequence, they represent only little or even no threat to e.g., local agriculture. On the other hand the results of our simple questionnaire, including information from the surveyed Polish wholesalers clearly showed that at least some alien taxa recorded by us can be widely distributed because of human activity, such as transport to many new locations (Fig. 1). Even if our data were based on information received from only two Polish wholesalers, it clearly suggests, that one season is needed to distribute such alien invertebrates throughout the entire country (in the case of Poland, it is an area of 312,679 km^2^). This is much faster comparing to the dispersal power of most alien terrestrial and freshwater invertebrates noted in Europe in the wild (Kappes & Hasse, 2012) but similar to species deriving from aquaria (Duggan, 2010; Chucholl, 2013; Kopecký, Kalous & Patoka, 2013; Maceda-Veiga et al., 2013; Patoka et al., 2015). We should also note that in Poland this type of hobby has not had such a long tradition as in Western Europe (especially in Germany, Belgium, the United Kingdom and the Netherlands) and it is not as popular as in countries mentioned above. As a result, the “dispersal power” of at least some of terrarium species listed by our study is even greater on the European scale.

Over 60 localities of alien terrestrial invertebrates noted during one season and located in the whole area of Poland mean dozens of new potential locations where those species can potentially be found in the wild in the near future. We do not provide data about their survival abilities in the wild in Poland (especially the winter period would especially be a natural limit for them), but at least one species from our list (*Oxidus gracilis*) was noted from the park areas placed close to greenhouses in Poland (Jędryczkowski, 1979; Jędryczkowski, 1982). Moreover, this exotic species was reported also from other regions of Europe, where it was found in the wild, e.g.: in Hungary (Korkós et al., 2002) and Bulgaria (Stoev, 2004). Additional alien species from our terrarium list were noted outside greenhouses in Germany, the Czech Republic, Italy (*Zonitoides arboreus*—Jueg & Von Proschwitz, 2003; Dvořak & Kupka, 2007; Evangelista et al., 2013) and Spain, Portugal, France (*Rhynchodemus sylvaticus*—Álvarez Presas et al., 2014). Molecular studies proved that one of the haplotypes of *Rhynchodemus sylvaticus* founded in Poland is the same as ones founded in Spain, Portugal and France by Álvarez Presas et al. (2014). Moreover, experiments made in Hungary with exotic Enchytraeidae indicated that some species noted mainly in greenhouses could survive the winter outside the greenhouse too (Boros, 2011).

Although most of the species noted by us in this study probably cannot survive in Poland outside terraria and/or greenhouses during winter because of low temperatures, we can expect that in case of future climate warming some species may have a greater chance of establishing in the future.

### Potential impact of alien terrarium invertebrates on nature

Most animal invasive species recorded in Europe are terrestrial invertebrates (Roques et al., 2009). Many of them have a significant influence on native flora and fauna as they are predators, plant eaters or vectors of different parasites and diseases (Williamson, 1996). Among the species found in our study at least one taxon is predatory—*Rhynchodemus sylvaticus* (the undetermined Chilopoda species is most probably also carnivorous). In the vivarium collections, nocturnal *R. sylvaticus* was observed hunting and feeding on flightless fruit flies (*Drosophila melanogaster*) and collembolans, invertebrates which are characterised by a quite high escape potential. This allows us to expect that if introduced into nature, it will be able to hunt a large variety of small epigeic invertebrates and it could present a potential risk to other species that have the same dietary needs, as was shown in cases of some other terrestrial planarians by Álvarez Presas et al. (2014).

Similar conclusions can be made in case of plant eaters, especially snails. These species were mentioned by us respectively as orchid and *Peperomia* spp. feeders, but records of the first one, from natural habitats in the Czech Republic (Dvořak & Kupka, 2007), Germany (Jueg & Von Proschwitz, 2003) and Italy (Evangelista et al., 2013) clearly suggest that it is a much more generalist feeder. It cannot be excluded that under preferable conditions *Zonitoides arboreus* will have a significant influence on some plant communities, as shown for many other alien terrestrial snails and slugs (e.g.: Kozłowski, 2012). Of course, in the case of all the mentioned alien terrarium species, we can expect that they can be a potential source of some parasites dangerous for native flora and fauna (e.g.: Diéguez-Uribeondo & Söderhäll, 1993).

Moreover, although actually we do not know if any of invertebrate taxa noted by us in vivaria can be found as potential vectors of any kind of diseases, generally it is known that many arthropods or snails are associated with different viruses, bacterias, and/or nematodes. In many cases such species are known as the phoretic (and parasitic) inhabitants of arthropods/molluscs but often are obligate and facultative plant/animal parasites too (e.g.: Hogenhout et al., 2008; Kanzaki & Giblin-Davis, 2018). As a result it cannot be excluded that alien invertebrates recorded in vivaria will have significant negative impact on native flora and/or fauna in the future, as it was suggested e.g., by Hogenhout et al. (2008) and Bragard et al. (2013) for other species.

## Conclusions

The list of alien invertebrates found in tropical vivarium husbandries includes species classified in different taxonomic groups, imported from different continents. It clearly shows the crucial role of human activity in this process. Although many of them would probably not be able to survive outside terraria and/or greenhouses, those which would acclimatise in new areas could present a potential risk to other species of the same dietary needs, as well as becoming important pests from the economic point of view. Moreover, their presence in habitats which are outside of their natural species ranges, may lead to a significant reduction of local biodiversity where they would be introduced.

##  Supplemental Information

10.7717/peerj.7617/supp-1 Supplemental Information 1 Localities and GPS coordinates of sites from which data on invertebrates were available

10.7717/peerj.7617/supp-2 Supplemental Information 2 Results of questionnaire

10.7717/peerj.7617/supp-3 Supplemental Information 3 Questionnaire used in the study

10.7717/peerj.7617/supp-4 Supplemental Information 4 DNA sequences

10.7717/peerj.7617/supp-5 Supplemental Information 5 Neighbor-joining tree based on COI K2p distance for sequences of Rhynchodemus sylvaticus and Rhynchodemus cf sylvaticus from Álvarez Presas et al. (2014) Platydemus monokwari was used as an outgroup. Arrows indicate sequences coming from this study. Numbers above branch represent bootstrap values.

10.7717/peerj.7617/supp-6 Supplemental Information 6 Neighbor-joining tree based on COI K2p distance for sequences of Trichorhina tomentosa (members of BIN ADG1585) Gammarus nekkensis was used as an outgroup (nearest neighbor pointed out by BOLD). Arrows indicate sequences coming from this study. Numbers above branch represent bootstrap values.

10.7717/peerj.7617/supp-7 Supplemental Information 7 Neighbor-joining tree based on COI K2p distance for sequences of Oxidus gracilis (members of BIN AAG5070) Sequence of Xystodesmidae was used as an outgroup (nearest neighbor pointed out by BOLD). Arrows indicate sequences coming from this study. Numbers above branch represent bootstrap values.

10.7717/peerj.7617/supp-8 Supplemental Information 8 Neighbor-joining tree based on COI K2p distance for sequences of Coecobrya tenebricosa (members of BIN ACZ2224) Sequence of Entomobryidae was used as an outgroup (nearest neighbor pointed out by BOLD). Arrows indicate sequences coming from this study. Numbers above branch represent bootstrap values.

10.7717/peerj.7617/supp-9 Supplemental Information 9 Neighbor-joining tree based on COI K2p distance for sequences of Zonitoides arboreus (members of all BINs) Sequence of Zonitoides nitidus was used as an outgroup. Arrows indicate sequences coming from this study. Numbers above branch represent bootstrap values.

10.7717/peerj.7617/supp-10 Supplemental Information 10 Neighbor-joining tree based on COI K2p distance for sequences of Guppya gundlachi (member of BIN ADH3853) and other members of family Euconulidae available from public resources of BOLD database Arrows indicate sequences coming from this study. Numbers above branch represent bootstrap values.

10.7717/peerj.7617/supp-11 Supplemental Information 11 Neighbor-joining tree based on COI K2p distance for sequences of Lehmannia valentiana (members of BIN AAJ1995) and other members of genus Lehmannia available from public resources of BOLD database Arrows indicate sequences coming from this study. Numbers above branch represent bootstrap values.

## Additional Information and Declarations

### DNA Deposition

The following information was supplied regarding the deposition of DNA sequences: GenBank Accession numbers for mt COI: MH295752–MH295766. BOLD dataset: TERIN (http://v4.boldsystems.org/).

### Data Availability

The following information was supplied regarding data availability: Specimens metadata is accessible through the public data set “TERIN” in the Barcode of Life Data Systems (http://v4.boldsystems.org/index.php/Public_SearchTerms). The raw data (results of questionnaire) are available as a Supplemental File.
