# Supplementary material for: Undesirable immigrants: hobbyist vivaria as a potential source of alien invertebrate species
Source: PeerJ. 2019 Sep 17;7:e7617. doi: 10.7717/peerj.7617 (PMC6753924; doi:10.7717/peerj.7617)
Supplement: Supplemental Information 1 [file peerj-07-7617-s001.docx]

| **No.** | **Locality** | **GPS co-ordinates** |
| --- | --- | --- |
| 1 | Bełchatów | N51.3690 E19.3566 |
| 2 | Będzin | N50.3256 E19.1254 |
| 3 | Białobiel | N53.1161 E21.5614 |
| 4 | Białystok | N53.1325 E23.1688 |
| 5 | Bielsko-Biała | N49.8277 E19.0601 |
| 6 | Branice | N50.0750 E17.8080 |
| 7 | Bukowiec | N51.7202 E19.6801 |
| 8 | Bydgoszcz | N53.1213 E18.0131 |
| 9 | Bytom | N50.3484 E18.9157 |
| 10 | Chróścina Nyska | N50.6228 E17.3667 |
| 11 | Cieszyn | N49.7998 E18.6355 |
| 12 | Częstochowa | N50.8120 E19.1204 |
| 13 | Ćmińsk | N50.9844 E20.5417 |
| 14 | Dziemiany | N54.0129 E17.7855 |
| 15 | Gdańsk | N54.3535 E18.6480 |
| 16 | Gdynia | N51.5489 E18.5305 |
| 17 | Gliwice | N50.2945 E18.6714 |
| 18 | Gorzów Wielkopolski | N52.7325 E15.2369 |
| 19 | Janikowo | N52.7538 E18.1133 |
| 20 | Janówka | N51.7491 E19.6939 |
| 21 | Jelenia Góra | N50.9044 E15.7194 |
| 22 | Kalisz | N51.7747 E18.0848 |
| 23 | Kielce | N50.8661 E20.6303 |
| 24 | Kluczbork | N90.9724 E18.2181 |
| 25 | Kłodawa | N52.2543 E18.9135 |
| 26 | Kozy | N53.3794 E15.4400 |
| 27 | Kraków | N50.0648 E19.9446 |
| 28 | Krapkowice | N50.4745 E17.9651 |
| 29 | Krynica-Zdrój | N49.4215 E20.9594 |
| 30 | Legnickie Pole | N51.1444 E16.2428 |
| 31 | Leśna | N51.0242 E15.2640 |
| 32 | Lębork | N54.5446 E17.7533 |
| 33 | Ligota | N51.7008 E16.6089 |
| 34 | Łódź | N51.7593 E19.4560 |
| 35 | Malbork | N54.0361 E19.0380 |
| 36 | Mikołów | N50.1790 E18.9038 |
| 37 | Nowa Sól | N51.8034 E15.7171 |
| 38 | Oława | N50.6751 E17.9213 |
| 39 | Opole | N51.7593 E19.4560 |
| 41 | Ostrowiec Świętokrzyski | N50.9295 E21.3852 |
| 42 | Oświęcim | N50.0344 E19.2098 |
| 43 | Otmuchów | N50.4661 E17.1734 |
| 44 | Pabianice | N51.6567 E19.3578 |
| 45 | Połaniec | N50.4330 E21.2807 |
| 46 | Poznań | N52.4064 E16.9252 |
| 47 | Późna | N51.8439 E14.6156 |
| 48 | Pszów | N50.0400 E18.3944 |
| 49 | Puławy | N51.4178 E21.9704 |
| 50 | Rąbień | N51.7903 E19.3239 |
| 51 | Reda | N54.6054 E18.3472 |
| 52 | Rybnik | N50.1022 E18.5463 |
| 53 | Rudawa | N50.1216 E19.7124 |
| 54 | Rzeszów | N50.0412 E21.9991 |
| 55 | Siedlce | N52.1690 E22.2900 |
| 56 | Siemianowice Śląskie | N50.3264 E19.0296 |
| 57 | Strzebiń | N50.6156 E18.8989 |
| 58 | Szczecin | N53.4284 E14.5535 |
| 59 | Ulan-Majorat | N51.8089 E22.4858 |
| 60 | Warszawa | N52.2327 E21.0157 |
| 61 | Wiązów | N50.8138 E17.2023 |
| 62 | Wrocław | N51.1089 E17.0481 |
| 63 | Zabrze | N50.3296 E18.7894 |
| 64 | Zamość | N50.7231 E23.2520 |
| 65 | Żory | N50.0447 E18.7006 |
