# Supplementary material for: Undesirable immigrants: hobbyist vivaria as a potential source of alien invertebrate species
Source: PeerJ. 2019 Sep 17;7:e7617. doi: 10.7717/peerj.7617 (PMC6753924; doi:10.7717/peerj.7617)
Supplement: Supplemental Information 2 [file peerj-07-7617-s002.docx]

Results of questionnaire placed on Polish internet forum (www.drzewolazy.pl)

| No. | Locality | Do you have any invertebrates in terrarium? | What was the source of plants used for decoration (if possible, please provide details about the city and/or country of plant origin |
| --- | --- | --- | --- |
| 1. | Bukowiec | yes | Germany |
| 2. | Warsaw | yes | Germany |
| 3. | Bydgoszcz | yes | - |
| 4. | Opole | yes | Germany, Czech Republic |
| 5. | Branice | yes | Germany, Czech Republic |
| 6. | Janówka | yes | - |
| 7. | Oświęcim | yes | Germany, Poland |
| 8. | Kielce | yes | - |
| 9. | Pabianice | yes | - |
| 10. | Siedlce | yes | - |
| 11. | Gdańsk | yes | Germany, Poland |
| 12. | Rudawa | yes | Germany, Poland |
| 13. | Kalisz | yes | Germany, Czech Republic |
| 14. | Bielsko-Biała | yes | Germany |
| 15. | Bełchatów | yes | Germany |
| 16. | Bełchatów | yes | Germany |
| 17. | Szczecin | yes | Germany |
| 18. | Warsaw | yes | Germany, Czech Republic |
| 19. | Zabrze | yes | Germany |
| 20. | Cracow | yes | Germany |
| 21. | Cracow | yes | Germany |
| 22. | Wrocław | yes | Germany, Poland |
| 23. | Łodź | yes | Germany, Czech Republic, The nederlands |
| 24. | Łodź | yes | Germany, Poland |
