# Supplementary material for: Undesirable immigrants: hobbyist vivaria as a potential source of alien invertebrate species
Source: PeerJ. 2019 Sep 17;7:e7617. doi: 10.7717/peerj.7617 (PMC6753924; doi:10.7717/peerj.7617)
Supplement: Supplemental Information 3 [file peerj-07-7617-s003.docx]

**Ankieta**

1/ Czy masz jakieś bezkręgowce w swoim terrarium? Jeśli tak,

2/ Jakie jest źródło roślin użytych przez Ciebie do wystroju terrarium (jeśli to możliwe , proszę, podaj szczegóły dotyczące miasta i/lub kraju ich pochodzenia)

**(and the English version of this questionnaire)**

**Questionnaire**

1/ Do you have any invertebrates in your terrarium? And if you do,

2/ What was the source of plants used for decoration (if possible, please provide details about the city and/or country of plant origin)?
