# Supplementary figures and images for: Undesirable immigrants: hobbyist vivaria as a potential source of alien invertebrate species
Source: PeerJ. 2019 Sep 17;7:e7617. doi: 10.7717/peerj.7617 (PMC6753924; doi:10.7717/peerj.7617)

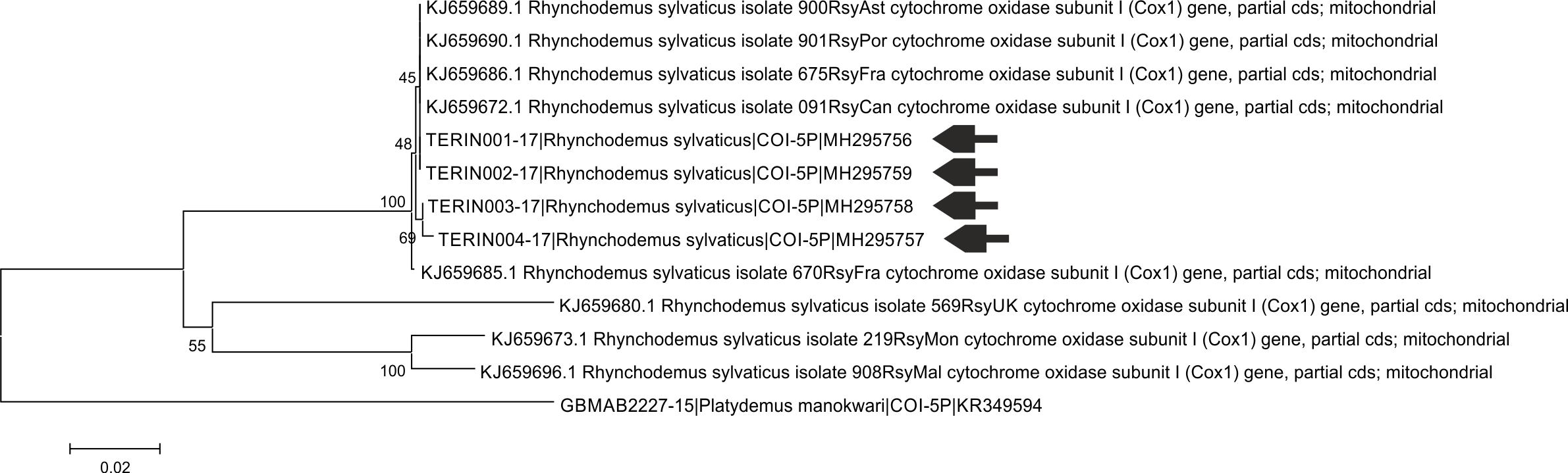

Supplement: Supplemental Information 5 — Platydemus monokwari was used as an outgroup. Arrows indicate sequences coming from this study. Numbers above branch represent bootstrap values. [file peerj-07-7617-s005.png]

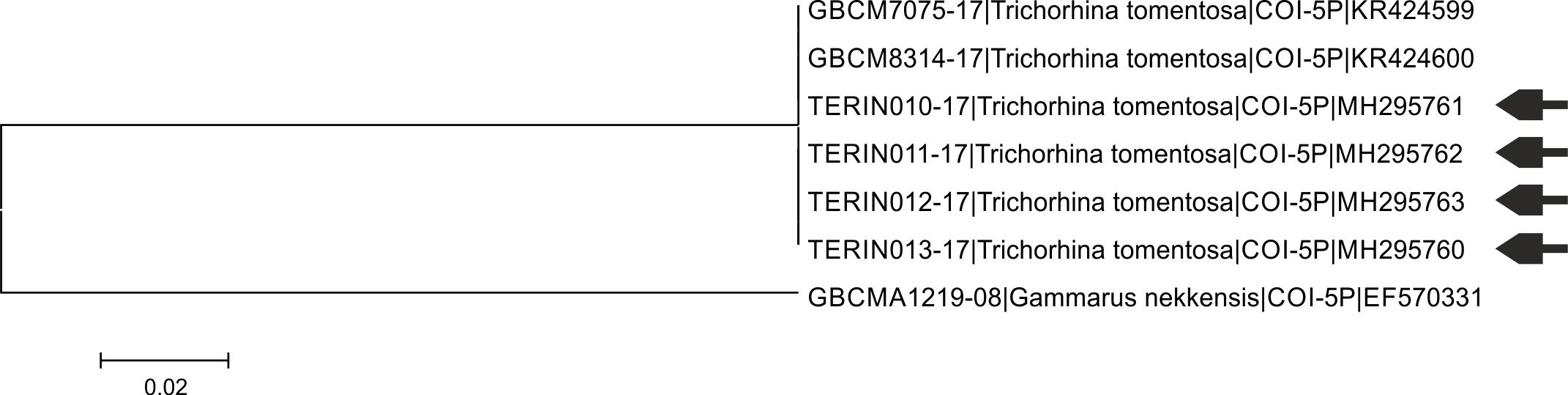

Supplement: Supplemental Information 6 — Gammarus nekkensis was used as an outgroup (nearest neighbor pointed out by BOLD). Arrows indicate sequences coming from this study. Numbers above branch represent bootstrap values. [file peerj-07-7617-s006.png]

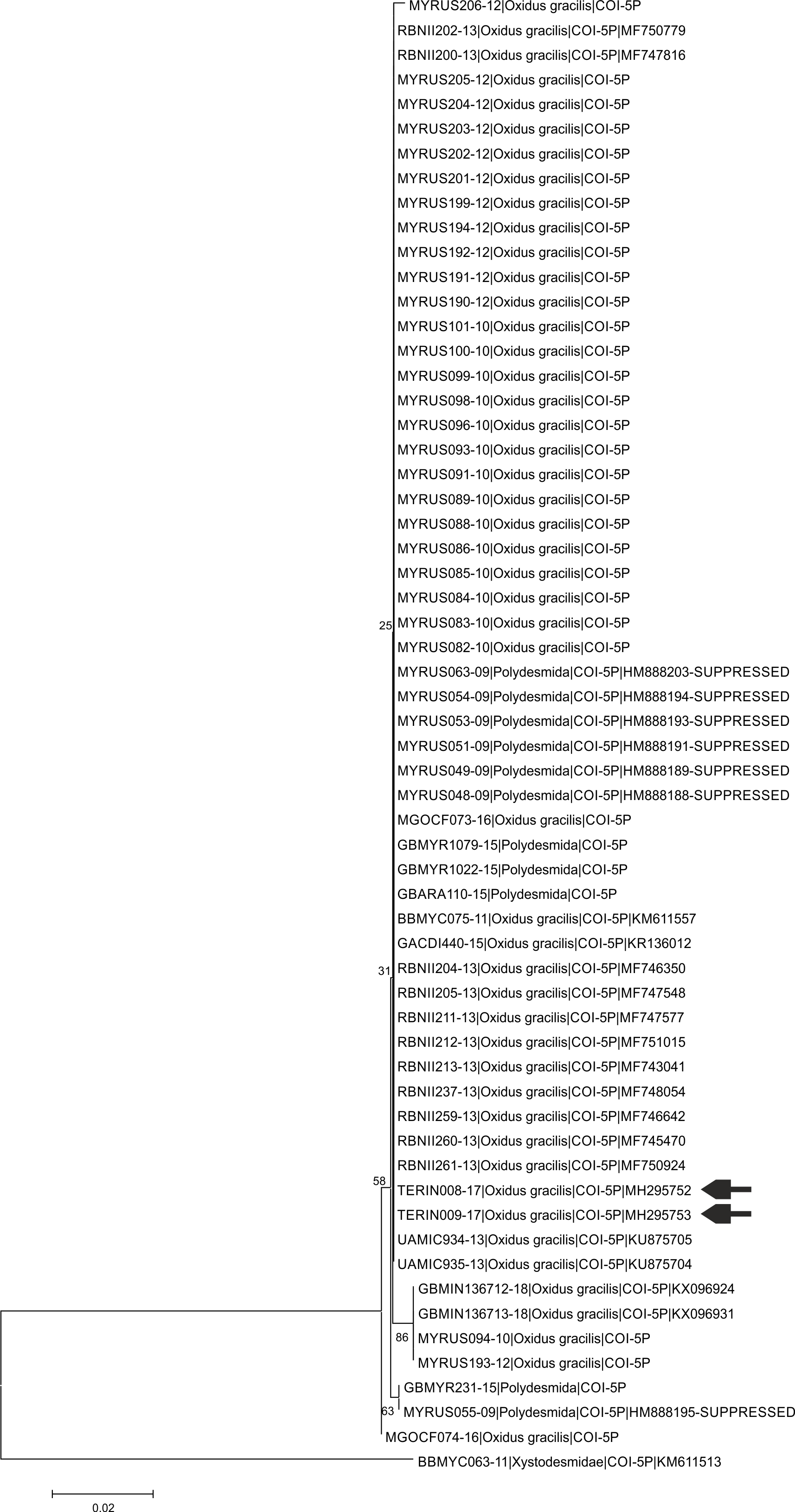

Supplement: Supplemental Information 7 — Sequence of Xystodesmidae was used as an outgroup (nearest neighbor pointed out by BOLD). Arrows indicate sequences coming from this study. Numbers above branch represent bootstrap values. [file peerj-07-7617-s007.png]

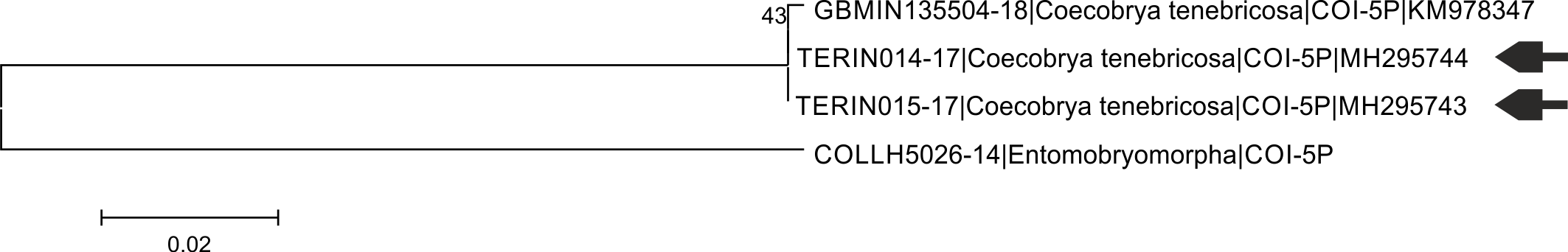

Supplement: Supplemental Information 8 — Sequence of Entomobryidae was used as an outgroup (nearest neighbor pointed out by BOLD). Arrows indicate sequences coming from this study. Numbers above branch represent bootstrap values. [file peerj-07-7617-s008.png]

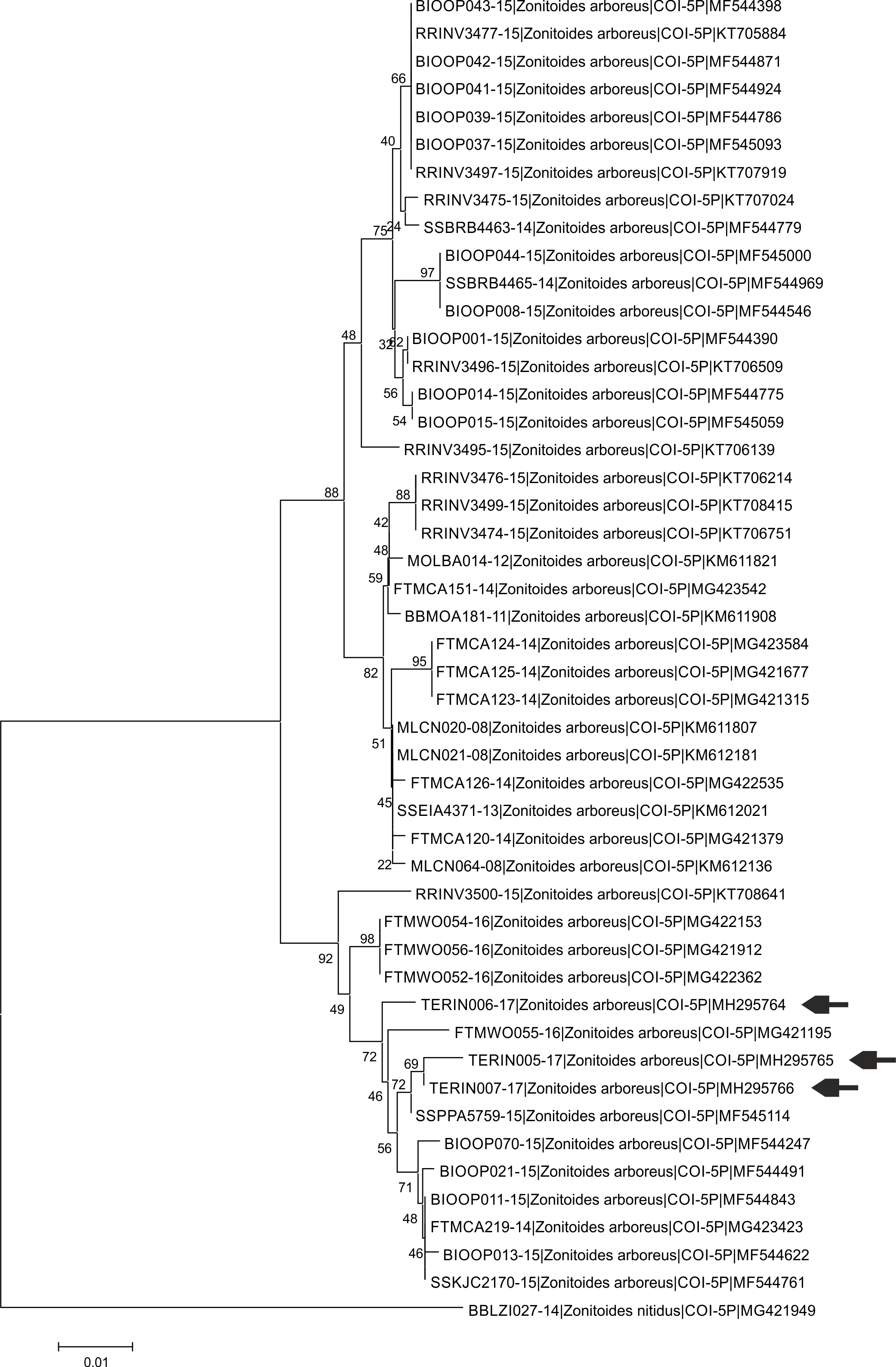

Supplement: Supplemental Information 9 — Sequence of Zonitoides nitidus was used as an outgroup. Arrows indicate sequences coming from this study. Numbers above branch represent bootstrap values. [file peerj-07-7617-s009.png]

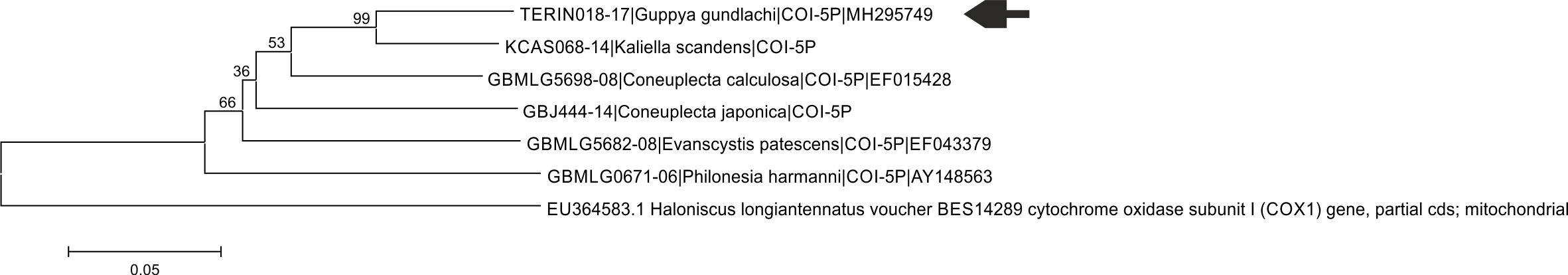

Supplement: Supplemental Information 10 — Arrows indicate sequences coming from this study. Numbers above branch represent bootstrap values. [file peerj-07-7617-s010.png]

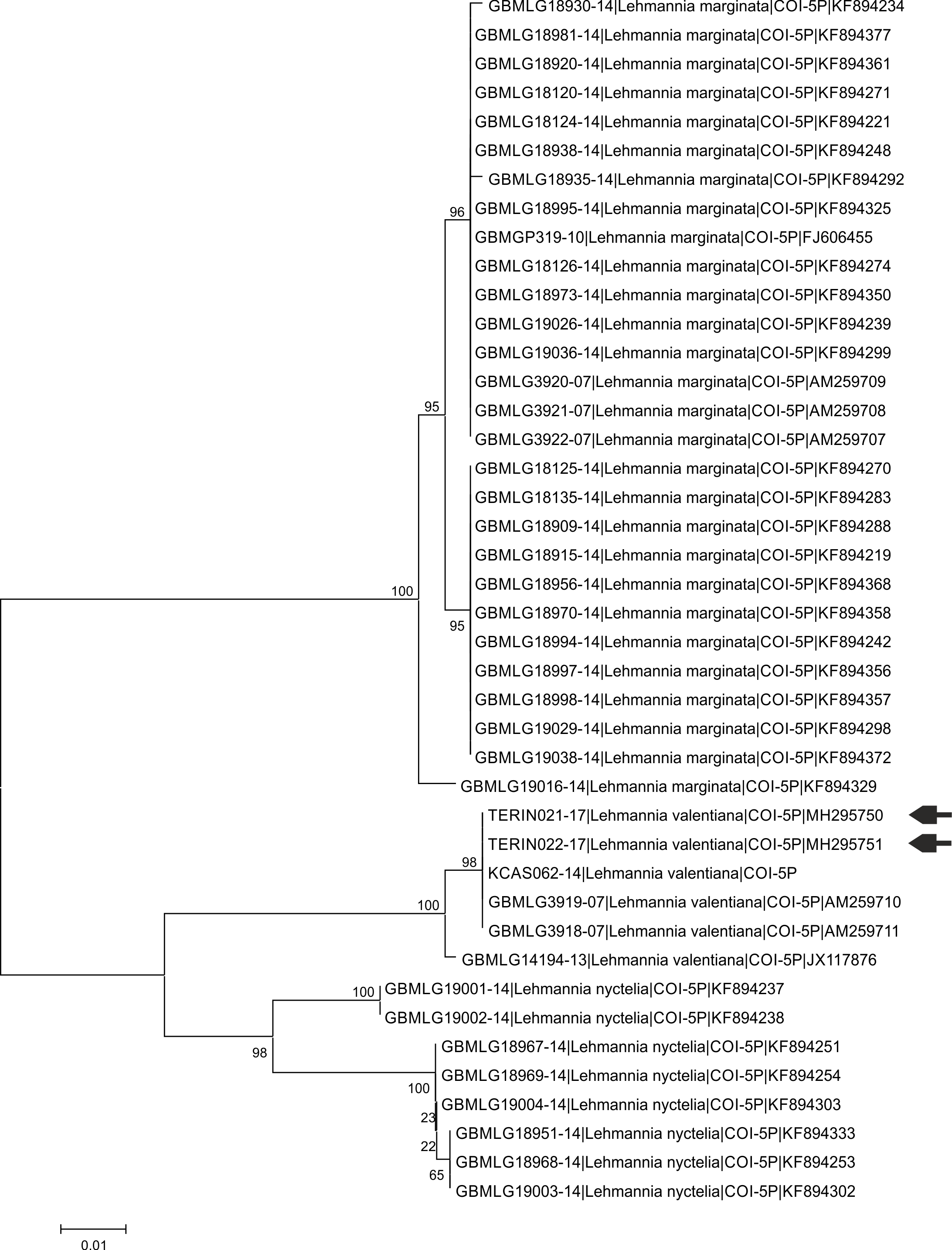

Supplement: Supplemental Information 11 — Arrows indicate sequences coming from this study. Numbers above branch represent bootstrap values. [file peerj-07-7617-s011.png]
